# Hydrochemical diversity of semi-natural water system on the background of environmental conditions

**DOI:** 10.1007/s10661-015-4555-x

**Published:** 2015-05-07

**Authors:** Adam Cudowski, Andrzej Stefan Górniak, Adam Więcko

**Affiliations:** Institute of Biology, Department of Hydrobiology, University of Białystok, Ciołkowskiego 1J, 15-245 Białystok, Poland

**Keywords:** Semi-natural water system, Water quality, Canal, Lake, River, Organic matter

## Abstract

Research carried out from 2007 to 2011 showed that a tested semi-natural water system with a diverse catchment (peat, peat-mineral and mineral) has a very varied quality of water, as evidenced by a the large range of conductivity (174–828 μS/cm) and pH (6.34–9.92). Natural lake parts of the semi-natural water system were similar to the artificial parts in terms of physico-chemical parameters, as evidenced by the lack of statistically significant differences between the water quality in both of these ecosystems. Taking into account the quality of various types of water in this semi-natural system, it can be seen that the river waters differed significantly (*p* < 0.001) in concentrations of biogenic elements (nitrogen, phosphorus) from another type of ecosystems. In addition, rivers poor in organic matter (TOC < 10.0 mg/L) which directly supplied the water system contributed to higher values of many tested parameters (EC, pH, TC, TN, SO_4_^2−^ or Cl^−^) in waters of the canal. In contrast, rivers with high values of concentrations of organic matter (TOC > 10.0 mg/L) contributed to a decline in values of those parameters. Moreover, lake waters within the tested semi-natural water system showed a “cleansing” function, because they caused a decrease in conductivity, pH or the concentration of carbon, total nitrogen, sulphate(VI) and chloride in waters of the canal.

## Introduction

The semi-natural water system consists of water reservoirs and/or lakes, artificial water canals and waters feeding the whole system. The anthropogenically transformed surface waters, intended to regulate the water cycle in the catchment or be used for transportation, can be defined as a canal (Gagg 1996). The characteristic feature of such systems is a small amplitude in water levels and low turbidity in the water column. Water flow in such semi-natural water systems is usually small and may be even unnoticeable in short sections connecting the reservoirs, which results in an increased sedimentation of seston and an abundant growth of aquatic vegetation. However, these naturally occurring processes in aquatic ecosystems may be disrupted by the intensive development of tourism (sailing, canoeing, etc.), which leads to re-suspension of sediments, increasing turbidity of waters and thus the reduced growth of plankton, periphyton and submerged vegetation. Previous studies, sometimes conducted on a global scale, have rarely dealt with the problem of the functioning of semi-natural ecosystems and the quality of their waters (biogeochemistry). The Augustów Canal, located in central Europe, is an appropriate example for the examination of hydrological and catchment conditions in shaping the chemical composition of the ecosystems of rivers and lakes in a temperate zone. The water systems of the Augustów Canal are amply suited as a kind of field laboratory, as they provide the possibility of examining the effect of different hydrochemical types of surface water mixing, on the biogeochemical processes occurring in those waters.

The aim of the study was to assess the spatial diversity of the physico-chemical composition of different types of surface water against catchment conditions. In addition, the task of this paper is to show the role of ecohydrological factors in the quality of river, lake and canal (artificial) waters in a semi-natural water system.

## Material and methods

### Study area

**Fig. 1:**
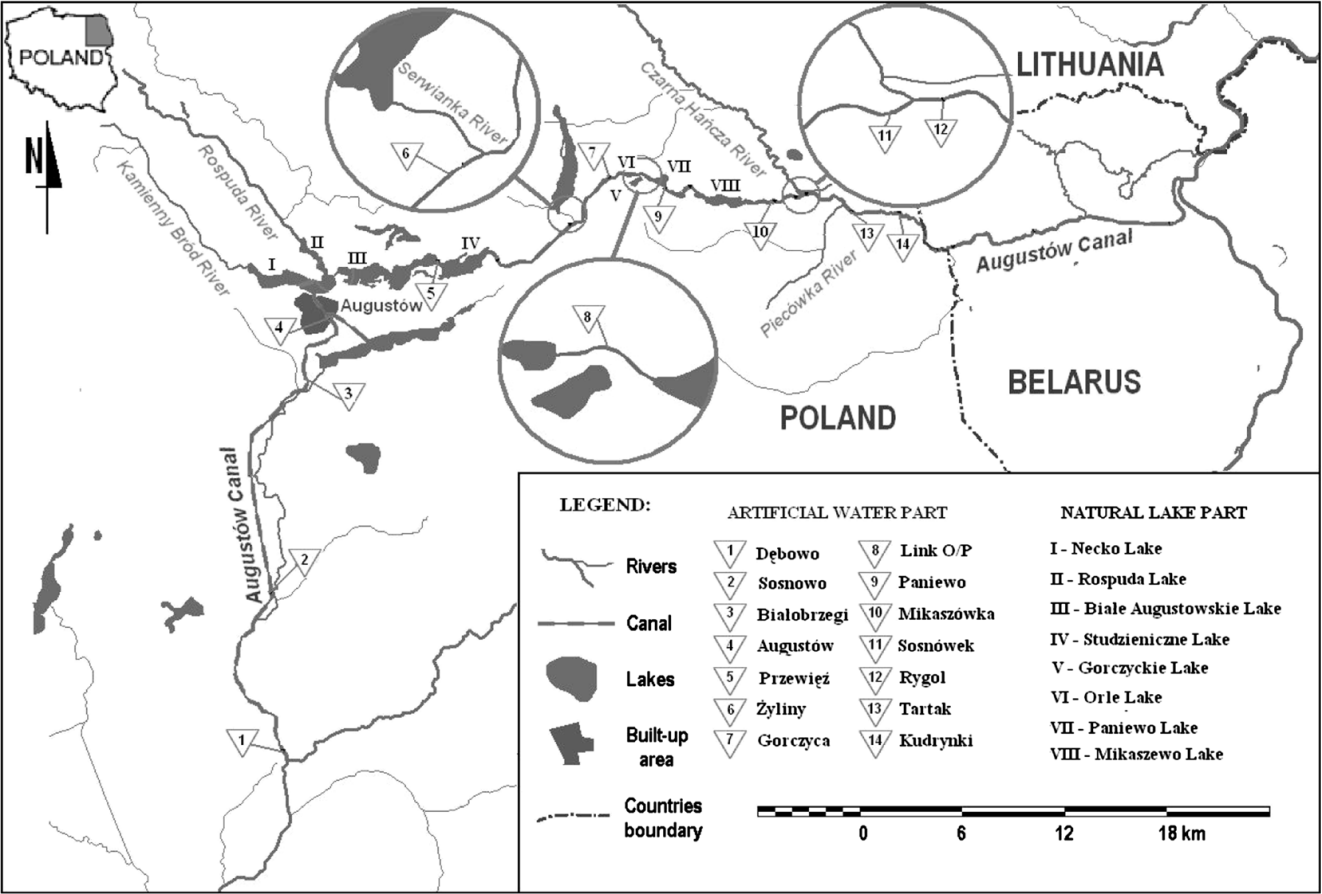
Location of the analysed stations on the semi-natural water system (Augustów Canal—NE Poland)

The Augustów Canal, located in central Europe, is a model object for research on the differences in physico-chemical quality of various types of surface waters (Fig. 1). This semi-natural water canal system is located in a catchment area of relatively diverse soil cover (Cudowski and Górniak 2008). The section of the system situated in the south of urban areas (Augustów City) is located in a peat catchment, while the remaining section is in a mineral catchment area. In the examined semi-natural water system, it was possible to distinguish natural river sections (rivers supplying the system) and natural water reservoirs (lakes) connected by artificial canals. Waters supplying the semi-natural water system have diverse contents of organic matter or pollution and different flow rates (Table 1). Supplies to the entire semi-natural water system are either direct, via rivers flowing directly into the canal, or indirect, via rivers flowing into lakes and then lakes supplying the water canal. The examined canal system includes eight natural lakes of different sizes, capacities and mictic type (Pietryczuk et al. 2014).

**Table 1:** Average ($\frac{\mathrm{minimum}-\mathrm{maximum}}{\mathrm{average}}$) velocity [m/s] and the streamflow quantity of river waters [m^3^/s] which supply semi-natural water system (*N* = 3) and the average amount of water [%] brought to the test system via the rivers

River	Czarna Hańcza	Serwianka	Rospuda	Piecówka	Kamienny Bród
V [m/s]	0.09–0.86	0.12–0.71	0.10–0.69	0.01–0.10	0.02–0.07
	0.51	0.68	0.58	0.08	0.04
SQ [m^3^/s]	6.54–8.01	1.13–2.02	7.03–9.44	0.11–0.98	0.09–1.03
	7.15	1.42	8.04	0.44	0.41
The amount of water brought to the canal via the rivers [%]	41	8	46	2.5	2.5

### Methods of analyses

Sampling of water was performed once a month from 2007 to 2011 at 22 designated positions along the route of the semi-natural water system and at 5 sites of water supplying it (Fig. 1). In the analysed period, about 1600 samples were collected. Samples were collected using a Limnos sampler at a depth of 0.5 m. In the field, we recorded the temperature and pH of the water and measured the electrolytic conductivity using a HQ40D Hach Lange probe. Furthermore, we measured the oxygen saturation and concentration in the water using a HQ10 Hach Lange oxygen probe. In addition, three times during the research, we measured velocity (V) and streamflow quantity (SQ) of river waters with an ADC digital current meter (OTT). Due to high air temperatures throughout May to September in the period of research, determinations of calcium, magnesium and bicarbonate ions were made in the field. In the remaining period, i.e. from October to April, analyses of these ions were performed in the laboratory. Bicarbonate was determined by acid-base titration. The concentrations of bicarbonate were converted to inorganic carbon (TIC). Calcium and magnesium were determined by complexometric method (APHA 2012). Other laboratory analyses were performed on the same day of sampling at the laboratory of the Department of Hydrobiology, University of Białystok, according to standard methods (APHA 2012). Nitrate(V), sulphate(VI) chloride, silicate, ammonium and reactive iron (DRFe) were determined using a Beckman DU-650 spectrophotometer. In addition, in the laboratory, we determined the concentration of dissolved organic carbon (DOC) by high-temperature catalytic combustion in a TOC-5050A analyser (Shimadzu), and particulate organic carbon (POC) was determined by chromate spectrophotometry (Bowman 1998). Sodium and potassium ions were determined by flame photometry (APHA 2012). Kjeldahl nitrogen (N_Kjel._) was determined using a Kjeldahl Tecator 2300 analyser in accordance with PN-EN 25633:2001. Chlorophyll *a* concentrations were determined by spectrophotometry (PN-86/C-05560/02). The concentration of individual fractions of phosphorus molybdate was determined by spectrophotometric method (APHA 2012). Total phosphorus (TP) was determined in non-filtered water subjected to UV mineralization, dissolved phosphorus (DP) in filtered water (0.45 μm) subjected to UV mineralization and reactive phosphorus (DRP) was determined in filtered water (0.45 μm).

Accepted physico-chemical data were statistically analysed according to the methodology presented by Griffiths (2008). The more than 35,000 results were subjected to statistical analysis. In order to investigate differences between the lakes, we used multivariate analysis—cluster analysis, where the Euclidean distance was adopted as a measure of probability, while Ward’s method was used for clustering. Principal components analysis (PCA) identified the variables that best explained the differences between the investigated objects and the absolute value of component loadings of individual characteristics allowed for their indication. The dispersion of a given characteristic was described based on the coefficient of variance (CV), defined as the ratio of the standard deviation from a given sample to its arithmetic mean, expressed as a percent value (Nairy and Rao 2003). For the interpretation of the relationship between environmental data (hydrological and physico-chemical parameters of rivers) and rivers with different concentrations of organic matter flowing through the different type of catchment, a redundancy analysis (RDA) was carried out. RDA is an enhancement of the commonly applied principal component analysis (PCA), but in contrast to PCA, RDA allows a direct analysis of the biotic environment components (terBraak 1994; van Wijngaarden et al. 1995). To test whether RDA analysis is appropriate for the dataset, the data were previously tested for normality (Kolmogorov–Smirnov test). DCA was used first to determine the character of variability in the studied assemblages: if the length of the first gradient is greater than 2 standard deviations, we can assume a unimodal variation; a length smaller than 2 SD indicates a linear variation (Lešp and Šmilauer 2003). The length of the first gradient for the fungi communities amounted to 1.71 SD, which indicated a linear variation, providing justification for the further use of a redundancy analysis. Relationships between nominal and quantitative variables were calculated with V-Cramera ranks, but relationships between quantitative variables were calculated with Pearson’s ranks.

## Results

**Fig. 2:**
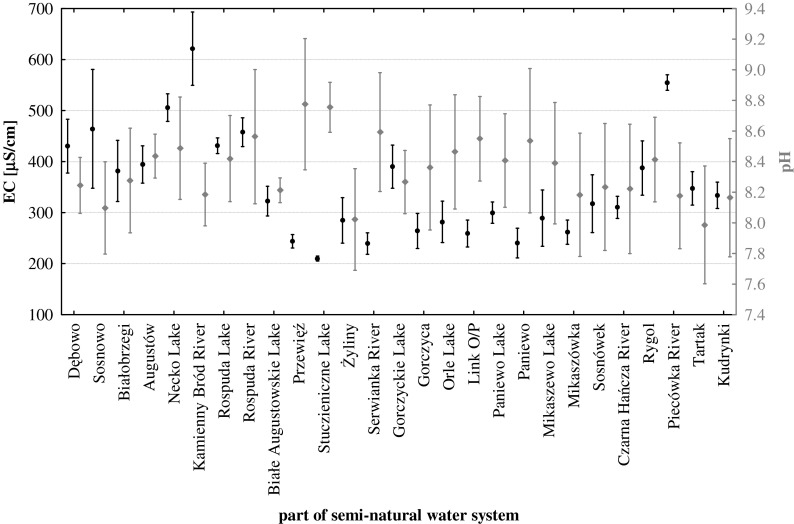
The variability of the mean electrolytic conductivity (EC) and pH in semi-natural water system (Augustów Canal—NE Poland)

**Fig. 3:**
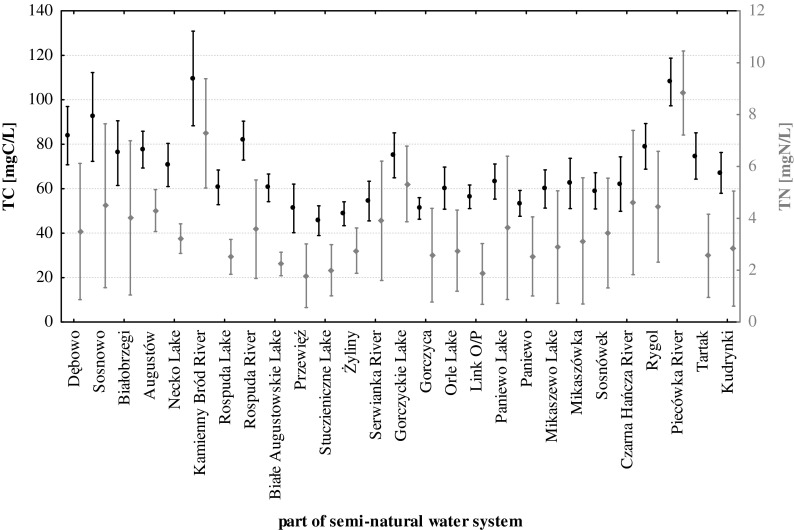
The variability of the mean concentrations of total carbon (TC) and nitrogen (TN) in semi-natural water system (Augustów Canal—NE Poland)

**Fig. 4:**
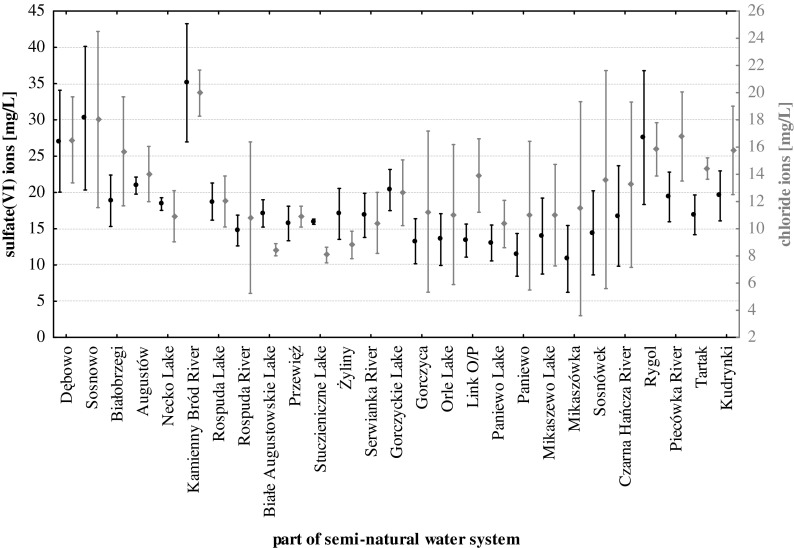
The variability of the mean concentrations of sulphate(VI) and chloride ions in semi-natural water system (Augustów Canal—NE Poland)

The water system of the Augustów Canal has a medium electrolytic conductivity at 337 ± 98.7 μS/cm, with a minimum of (174 μS/cm) in the central section of the system (Serwianka River) and a maximum (828 μS/cm) in the anthropogenically contaminated river (Kamienny Bród) (Fig. 2). The highest values for this parameter were typically found in the natural river sections, while the lowest were found in the natural lake sections (Table 2). The average pH of the examined water system was 8.32 ± 0.44; the minimum (6.34) was observed in a river section rich in organic matter (Piecówka River) and a maximum (9.92) in the lake with an elevated concentration of organic matter (Rospuda Lake) (Fig. 2). The most acidic waters belonged to the natural river part of the canal, with the most basic reaction was characteristic of the natural lake part (Table 2). The maximum concentrations of both total carbon (120.3 mgC/L) and total nitrogen (15.4 mgN/L) were found in an anthropogenically contaminated river (Kamienny Bród River) and the minimum levels in a lake with a low content of organic matter (Studzieniczne Lake), in which total carbon concentrations reached 23.3 mgC/L and total nitrogen 0.34 mgN/L (Fig. 3). Both in the case of sulphate(VI) and chloride, concentration maxima were found in the Kamienny Bród River (Fig. 4) 48.3 mg/L and 33.1 mg/L, respectively. The minima were found in the artificial part of the system (Link O/P) 2.69 mg/L and 3.84 mg/L. It should also be noted that parameters such as total organic and inorganic carbon, calcium, magnesium, sulphate(VI), chloride and silicate reached maximum concentrations in the natural river part of canal, while minima were found in the natural lake part, with the exception of sulphate(VI) and chloride, whose minimum concentrations were found in artificial sections (Table 2). In the case of nitrate(V) and nitrate(III) and the fractions of phosphorus (TP, DP, DRP) and total organic nitrogen (TON), the minimum concentrations of these parameters were found in the natural river part of the canal, while the maxima were found in the artificial sections (Table 2). The concentrations of ammonium, sodium and potassium ions were minima in the artificial part of the system and maxima were found in the natural lake part (Table 2). It should be noted that the studied system is fed by rivers with different streamflow quantities and velocities (Table 1), pollution levels and different organic matter content (Table 3).

**Table 2:** Physico-chemical parameters of water quality of different types of surface ($\frac{\mathrm{minimum}-\mathrm{maximum}}{\mathrm{average}}$) on the example of the semi-natural water system

Parameter [unit]	Natural lake part	Natural river part	Artificial water part
EC [μS/cm]	215–560	174–828	222–641
	294	359	314
pH	7.45–9.92	6.34–7.94	6.71–8.02
	8.09	7.51	7.54
Temperature [°C]	0.3–27.4	0.9–25.3	0.5–25.1
	11.3	10.2	10.4
Oxygen [mg/L]	3.9–12.3	5.2–12.8	4.1–11.1
	8.8	9.4	8.8
SWWT [%]	44.7–125	52.6–127	45.9–120
	94.5	98.8	95.5
TIC [mgC/L]	21.1–61.2	28.7–80.9	21.8–59.8
	37.4	41.7	38.4
Ca^2+^ [mg/L]	31.9–77.8	39.9–87.8	33.4–80.2
	51.1	62.3	53.3
Mg^2+^ [mg/L]	1.1–45.9	1.8–55.7	1.2–44.9
	6.9	10.1	9.4
TOC [mgC/L]	2.2–34.2	1.7–39.4	2.4–33.1
	14.1	15.5	14.5
NO_3_^−^ [μgN/L]	25.4–584	6.24–628	7.1–875
	195	101	214
NO_2_^−^ [μgN/L]	5.3–21.9	4.3–11.2	5.6–34.9
	7.6	5.4	10.2
NH_4_^+^ [μgN/L]	61.3–511	41.3–427	30.1–298
	199	142	117
TON [mgN/L]	0.2–10.1	0.1–14.5	0.2–15.1
	1.8	1.0	1.9
TP [μgP/L]	51.3–625	41.7–447	59.9–975
	190	99.8	201
DP [μgP/L]	40.9–347	40.6–318	56.3–489
	101	44.1	159
DRP [μgP/L]	1.9–90.4	1.8–88.7	24.9–178
	23.5	10.3	88.2
DRFe [μg/L]	36.2–572	27.9–496	41.0–437
	144	102	96.4
SiO_4_^4−^ [mg/L]	0.1–4.6	0.4–6.2	0.2–3.3
	0.9	1.9	1.1
SO_4_^2−^ [mg/L]	9.4–30.5	10.1–48.3	2.7–40.1
	18.4	26.9	22.4
Cl^−^ [mg/L]	2.2–29.8	5.1–33.1	3.8–25.8
	11.2	14.1	11.7
Na^+^ [mg/L]	0.5–7.4	0.2–6.4	0.1–5.6
	2.5	2.3	1.5
K^+^ [mg/L]	0.2–5.9	0.2–5.7	0.1–5.0
	1.2	1.1	0.8
Chlorophyll *a* [μg/L]	0.9–27.8	0.2–14.2	0.1–20.5
	7.6	4.2	6.5

**Table 3:** Physico-chemical parameters of water quality of selected fresh water ecosystems ($\frac{\mathrm{minimum}-\mathrm{maximum}}{\mathrm{average}}$) with different organic matter content

Type of water	Water poor in organic matter			Water rich in organic matter		
	River	Lake	Lock	River	Lake	Lock
Position	Czarna Hańcza	Białe Augustowskie	Paniewo	Piecówka	Gorczyckie	Sosnowo
EC [μS/cm]	306–401	213–394	213–311	307–628	192–403	209–513
	323	334	257	548	381	474
pH	7.59–9.52	8.01–8.84	7.98–8.95	6.34–8.42	7.42–9.27	7.53–8.99
	8.23	8.24	8.57	8.17	8.36	8.12
Temperature [°C]	1.1–20.7	0.9–23.1	0.8–23.9	1.3–19.8	0.7–21.9	0.7–22.1
	10.6	9.9	9.8	11.7	11.2	10.1
Oxygen [mg/L]	4.3–15.7	6.8–12.1	6.1–10.8	6.2–16.2	6.1–10.5	7.2–11.8
	9.9	9.8	9.5	9.1	8.2	9.2
SWWT [%]	54.2–123	65.9–119	66.3–123	65.1–124	52.0–122	76.5–114
	94.6	99.7	98.5	92.3	91.8	93.7
TIC [mgC/L]	37.4–70.6	31.3–66.9	37.8–58.4	47.4–117	29.5–57.4	31.3–58.2
	53.5	49.0	46.8	83.0	59.6	76.5
Ca^2+^ [mg/L]	36.1–89.6	39.2–62.7	33.1–69.9	37.1–104	36.1–67.1	28.2–71.2
	66.9	49.8	52.3	76.6	53.2	55.1
Mg^2+^ [mg/L]	1.7–50.7	1.2–37.5	3.9–42.7	2.7–31.2	2.1–50.2	1.3–29.2
	23.1	6.9	8.3	24.2	9.8	9.8
TOC [mgC/L]	4.9–23.1	7.0–18.8	5.9–17.4	9.1–44.3	6.8–31.2	5.2–33.1
	8.6	9.5	9.6	27.2	18.3	19.1
NO_3_^−^ [μgN/L]	49.1–459	8.0–588	20.1–413	94.8–1101	14.9–984	6.9–868
	240	197	217	988	611	625
NO_2_^−^ [μgN/L]	4.3–10.1	6.6–21.9	5.6–10.6	5.3–11.2	5.3–10.5	6.3–34.9
	8.9	5.3	6.3	11.5	7.9	8.2
NH_4_^+^ [μgN/L]	44.0–376	79.4–488	69.4–393	65.6–565	74.7–433	89.5–396
	224	187	181	287	194	216
TON [mgN/L]	0.2–10.8	0.3–9.6	0.2–4.8	0.4–10.0	0.2–6.8	0.3–5.1
	3.3	1.8	2.1	6.1	3.7	3.9
TP [μgP/L]	34.5–583	31.2–379	27.4–351	46.7–524	58.7–588	41.5–284
	115	150	144	171	231	179
DP [μgP/L]	34.6–248	33.5–211	23.6–764	29.5–234	48.2–251	56.9–273
	96.2	50.6	52.4	140	101	98.2
DRP [μgP/L]	11.1–199	1.1–89.2	0.8–72.6	4.4–160	3.6–23.2	1.0–152
	39.8	21.7	23.5	19.1	11.5	10.2
DRFe [μg/L]	41.8–471	37.8–282	40.4–420	34.0–415	41.3–158	49.8–175
	152	183	177	76.1	84.9	82.2
SiO_4_^4−^ [mg/L]	0.8–5.2	0.1–1.2	0.3–2.7	1.1–5.8	0.1–4.2	0.8–4.3
	1.8	0.5	0.7	3.1	2.1	1.7
SO_4_^2−^ [mg/L]	10.1–26.9	11.5–18.9	10.2–19.3	11.7–44.3	12.3–24.8	8.1–36.2
	17.4	17.4	12.6	19.8	20.3	30.3
Cl^−^ [mg/L]	8.4–40.9	6.2–34.3	7.8–47.8	7.9–29.9	6.1–37.3	7.8–32.3
	13.3	8.9	10.1	16.6	12.6	18.2
Na^+^ [mg/L]	0.9–5.1	1.3–6.8	1.6–3.8	1.0–4.7	0.7–6.8	1.3–8.4
	2.3	2.8	2.9	3.3	4.9	5.7
K^+^ [mg/L]	0.1–1.8	0.1–2.4	0.2–1.5	0.2–2.9	0.1–5.1	0.2–3.4
	0.9	0.9	0.8	1.4	2.6	2.7
Chlorophyll *a* [μg/L]	0.9–18.9	2.1–24.2	0.6–25.5	0.7–38.2	0.9–50.3	1.3–40.2
	11.5	17.3	18.7	21.4	31.6	30.3

Analysing the studied system for the content of organic matter, it should be noted that regardless of the type of freshwater ecosystem, water rich in organic matter showed higher concentrations of virtually all studied physico-chemical parameters in comparison with waters poorer in organic carbon compounds (Table 3). We recorded elevated concentrations of a number of parameters tested in the waters located in the south of the city of Augustów (Figs. 3 and 4). There were two exceptions: water poorer in organic carbon compounds had higher values of dissolved reactive phosphorus and dissolved reactive iron (DRFe) compared to waters with elevated concentrations of organic matter (Table 3).

## Discussion

**Fig. 5:**
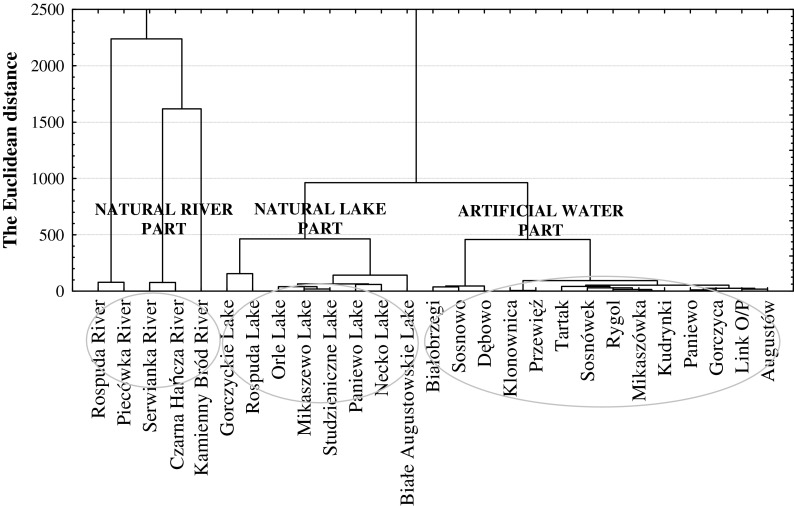
Result of the cluster analysis of semi-natural water system (Augustów Canal—NE Poland)

**Fig. 6:**
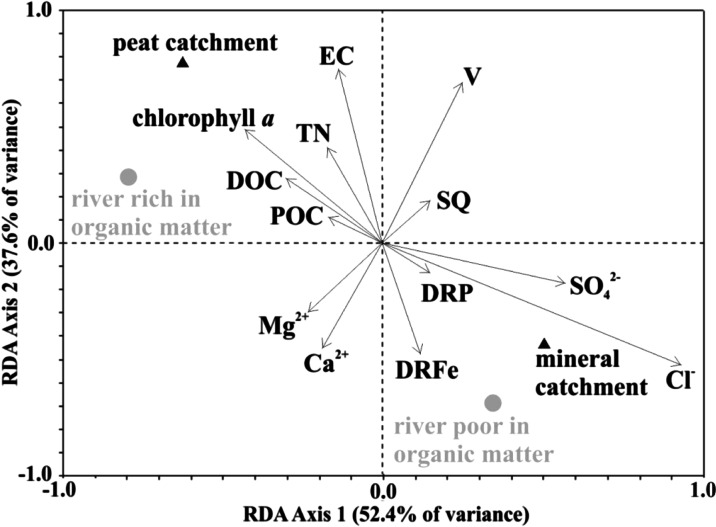
Triplot of redundancy analysis (RDA) integrating type of catchment, environmental variables (hydrological and physico-chemical parameters of rivers) and type of river with different concentrations of organic matter. The measured environmental variables are shown as *arrows*. The rivers with different concentrations of organic matter flowing through the different type of catchment are placed as perpendicular projections of variable vectors according to the environmental conditions measured at each river and different type of catchment. The vector orientations represent the direction of strongest change; vector lengths represent relative importance. In the RDA analysis, a positive correlation between two environmental factors is expressed by relatively *long vectors* pointing approximately in the same direction, whereas a negative correlation is indicated by *arrows* pointing in opposite directions. The longer the environmental cline, the stronger the relationship of that parameter with the community. *Perpendicular arrows* indicate no correlation

Hydrochemical variation of water quality in the examined semi-natural system is comparable with research on the Taldanda Canal by Samantray et al. (2009) and El-Salam Canal by Mohamed (2013). Pollution of this system waters, e.g. manifested in high conductivity, was comparable to the Boca Raton canal system (McKenzie 1995), although much less than the Sanganur (Kumari et al. 2006) and El-Khashaba canals (Abdalla and Scheytt 2012). On the basis of the measured physico-chemical composition of the semi-natural water system, we performed a cluster analysis which confirmed its hydrochemical division into three parts: a natural river, a natural lake and an artificial lake (Fig. 5). A natural lake part of the system included lakes with a low content of organic carbon (TOC < 10.0 mgC/L) and lakes with a high organic matter content (TOC > 10.0 mgC/L) (Table 3). This division has been made according to Dunalska (2011), who makes the fertility of the waters dependent on the organic matter concentrations. Hydrochemical variation in the artificial part was mainly due to the diversity of the canal’s catchment (Cudowski and Górniak 2008) which could be separated into two parts (Fig. 5). The first is located in the south of the urban areas (Augustów City), which runs through a peat catchment, whereas the other flows through a peat-mineral catchment, located in the east of Augustów (Cudowski and Górniak 2008). The performed analysis of the physico-chemical properties of waters showed that phosphorus and nitrogen compounds were the most differentiated throughout the system (Table 2). This was confirmed by statistically significant differences (*p* < 0.001) in the concentrations of phosphorus (TP, DP, DRP) and nitrogen (NO_3_^−^, NO_2_^−^) between the natural river part and the other parts of the tested ecosystem. We found almost no statistically significant differences between natural lakes part and artificial water part, with the only exception of inorganic nitrogen and phosphorus (*p* < 0.001) (Table 2). Our study showed that the direct catchment area of the tested system had a great influence on the quality of water (Fig. 6). Abundance of both mineral and organic compounds in the southern sections of the system was the result of land use in the catchment area (arable fields and meadows) and the peat nature of the catchment (Peterjohn and Correll 1984). Elevated concentrations of dissolved reactive iron and phosphorus in the studied waters were due to the fact that they flowed through the mineral catchment, which is confirmed by a significant statistical dependence at *p* < 0.001 (Fig. 6). Such water has very low concentrations of organic carbon, and so neither phosphorus nor iron can create complexes with organic substances (Wetzel 2001).

Using the hydrochemical classification of surface waters by Altowski and Szwiec (1956), we found that waters supplying the examined river–lake system belong to bicarbonate-calcium-magnesium type (HCO_3_^−^-Ca^2+^-Mg^2+^), similar to other waters in this climate zone, for example in the Tatra National Park (Żelazny et al. 2013). This type differs from river waters in other climatic zones, e.g. in India with calcium-sodium-bicarbonate (Manoj et al. 2013) or calcium-magnesium-chloride types (Rama et al. 2013). Multidimensional cluster analysis showed three groups of rivers supplying the waters of the studied system. The first group included clean rivers with a relatively low organic carbon content (TOC < 10.0 mgC/L), the second group comprised clean rivers with a high content of organic matter (TOC > 10.0 mgC/L), while the third group included anthropogenically polluted river. The electrolytic conductivity (Fig. 2) of that contaminated river greatly exceeded the geochemical background value in this part of Poland (Table 2) but was close to waters of the Biebrza River (Grabowska et al. 2014) or the Vistula River (Napiórkowski and Napiórkowska 2013). River waters supplying the semi-natural system had varied mineral concentrations of dissolved solids, which were reflected in the average electrolytic conductivity (*p* < 0.001) with the lowest and the highest conductivity of the rivers differing by 40 % (Fig. 2). Those rivers that flowed through mineral catchment areas used for agricultural purposes were rich in nutrients (Fig. 6), although in the case of the Kamienny Bród River, the reported concentrations of nitrogen were about two orders of magnitude lower than in the Mahanadi River and Atharbanki River (Samantray et al. 2009), which shows the relatively good state of this Polish river. High concentrations of nutrients provide excellent conditions for the development of algae (Fig. 3), which has been confirmed by studies on the Narew River (Poland) (Grabowska and Mazur-Marzec 2011; Grabowska 2012). Increased concentrations of phosphorus in the Piecówka River were partly due to supply from rural areas and partly to drainage of catchments covered by soils rich in organic matter. Waters supplying the tested river–lake system bring organic matter (both dissolved and suspended), which has a great effect on the processes occurring in this water systems. Rivers with a low content of organic matter (e.g. Czarna Hańcza River) caused a statistically significant (*p* < 0.005) increase in average conductivity in the waters of the whole system (Fig. 2), in contrast to the uncontaminated rivers with increased concentrations of organic matter (Fig. 6). This regularity can be explained by the possibility of mineral complexation with organic matter. Semi-natural water system with elevated total carbon and nitrogen result from the low levels of organic carbon, and a decrease in both parameters results from the inflow of waters with higher values of organic carbon (Fig. 2). This can be explained solely by the influence of organic matter on the biogeochemical processes occurring in the tested system. The streamflow quantity of water flowing into the canal and its velocity had no effect on the quality of the waters (Fig. 6) and did not significantly modify the physico-chemical composition of the canal water. Rivers with different velocities (Table 1) and a low content of organic matter (Czarna Hańcza and Serwianka) always equally influenced the quality of the river-like system waters (*p* < 0.05) by increasing the concentrations of a number of physico-chemical parameters (Figs. 2, 3 and 4). Conversely, rivers with different velocities (Table 1) and with a high content of organic matter (Piecówka and Rospuda) always changed the water quality of lakes (*p* < 0.05) by decreasing concentrations of a number of physico-chemical parameters in the tested system (Figs. 2 and 4). The effect of both types of the river on the concentration of sulphate(VI) or chloride (Fig. 4) in the waters of the river–lake system was the same as for carbon or nitrogen. On this basis, it can be concluded that a river rich in organic matter may to some extent control the anthropogenic pollution of waters via complexation processes (Wetzel 2001). Summarizing, in the examined river–lake system, rivers with a low amount of organic carbon compounds may increase the amount of dissolved organic and mineral compounds, with a consequent increase in electrolytic conductivity in the water (Fig. 2). Rivers rich in organic carbon decrease many physico-chemical parameters of the water system. These processes and regularities, which take place in this open, dynamic system, are consistent with the theory of Le Chatelier-Braun; according to whom if a system is exposed to an external stimulus, then the system responds to counteract the stimulus (Müller 1884).

The multivariate cluster analysis placed the tested lentic waters, which are an essential part of semi-natural water system into two groups: (i) hydrochemical group comprising lakes with relatively high concentrations of organic matter and nutrients (Gorczyckie and Rospuda Lakes) which can be classified as eutrophic waters and (ii) mesotrophic lakes with low concentrations of organic substances. Principal component analysis showed that the first three principal components explain about 91 % of the variation between the lakes, of which the first component explains 46 %. Chemical parameters (characteristics of factor I) showed the difference between the studied lakes were carbon, nitrogen and phosphorus compounds, which play a key role in biological processes in aquatic ecosystems. Groups of features grouped in factors II and III explain approximately 22 % of the variation between the lakes. In connection with sufficient light, appropriate temperature or oxygen concentration, they allow for increased phytoplankton growth, resulting in an increase in the concentration of chlorophyll *a* (these parameters are included in the second component). The third component combines water pH with the concentration of reactive iron whose form significantly depends on pH (Table 4). Hydrochemical analysis of the natural lake part of the water system showed that the concentration of calcium ions in the studied lakes was characterized by high variability (CV = 367 %), and its growth was accompanied by a decrease in the concentration of dissolved reactive phosphorus, as confirmed by the negative correlation (*r* = 0.88, *p* < 0.001) between these parameters. This is due to the fact that the higher the concentration of calcium ions in the water, the faster their co precipitation with phosphorus and sedimentation to the sludge. That is why the process of lake eutrophication is slowed down. The obtained concentrations of orthophosphate(V) ions in the studied lakes (Table 2) were still relatively high, e.g. compared with lakes in the USA, where in the waters of the chain of lakes in Minneapolis, Minnesota (Huser et al. 2011), total phosphorus concentrations were only half higher than the dissolved reactive phosphorus in the examined lakes of the river–lake system in Poland. In our study, high concentrations of chlorophyll *a*, directly associated with the intense proliferation of algae in lake waters rich in organic matter, were accompanied by very low concentrations of DRP and ammonium nitrogen, which has also been observed by Freeman (2002) and Fried et al. (2003). In addition, we observed relatively low concentrations of inorganic carbon at high concentrations of chlorophyll *a* in water. This inverse relationship (*r* = 0.91, *p* < 0.01) was due to the fact that at high pH values of inorganic carbon becomes a factor inhibiting the growth of algae (Jaworski et al. 1981). In turn, low concentrations of chlorophyll *a* in the mesotrophic lakes of the tested system were caused by a low N:P ratio of 2.5, because nitrogen can be a major factor limiting the growth of algae (Harpole et al. 2011). At the same time, the lakes had high concentrations of DRFe, as these waters are characterized by low organic matter content and thus the possibility of complexation with organic compounds was limited (Wetzel 2001). Low concentrations of silicate ions in mesotrophic lakes are caused by an intensive development of diatoms, yellow algae and other algae species, which incorporate silicon in their own biomass. This is supported by the significant statistical correlation (*r* = 0.89, *p* < 0.005) we found between the concentration of chlorophyll *a* and silicate ions in the water. This pattern also results from the fact that polysilicon acid, found in the waters in the form of colloids, is used for the synthesis of proteins, carbohydrates or chlorophylls (Exley et al. 1993). As shown by our results, waters of the lakes of the studied semi-natural water system change the hydrochemical composition of the canal, improving the quality of its waters. This occurs e.g., by processes of calcium carbonate precipitation, a consequence of intensive growth of algae. Consequently, this leads to a deficit of carbon(IV) monoxide in water; in order to preserve the balance in water, part of bicarbonate is precipitated (Brezonik and Arnold 2011).

**Table 4:** Coordinates factor and the percent of cumulative explained variation of principal component analysis based on selected physico-chemical parameters of the lakes water quality of the semi-natural water system

Variable	Factor					
	I	II	III	IV	V	VI
EC	−0.3474	0.0178	0.4275	−0.5232	*0.7024*	−0.2161
pH	0.2147	−0.3347	*−0.7124*	0.1145	0.1796	−0.1128
Temperature	0.1007	*0.6547*	−0.2482	0.0147	0.2178	0.0145
Oxygen	−0.1278	*0.8423*	0.0977	0.1487	0.1244	−0.1348
Chlorophyll *a*	−0.2224	*0.8123*	−0.0097	0.3012	−0.2414	0.0997
TIC	*0.6875*	−0.1127	0.3782	−0.1124	−0.0006	0.2478
TOC	*0.9021*	−0.0545	−0.2486	0.0249	−0.1247	0.1247
TIN	*0.7496*	−0.1746	−0.3542	−0.4024	0.2476	0.4597
TON	*0.7054*	0.0874	0.4269	0.3784	0.4478	0.4111
SO_4_^2−^	0.2144	0.2991	0.0019	*−0.8687*	0.2179	−0.1991
Cl^−^	0.4126	−0.2004	0.2246	*−0.8824*	−0.0056	−0.3346
DRP	*−0.8012*	0.5106	−0.1899	−0.1241	0.3778	0.1249
DRFe	−0.2145	−0.0479	*−0.8934*	0.0845	0.4273	−0.1864
SiO_4_^4−^	0.2473	−0.0441	0.0878	0.0966	−0.0871	*−0.8436*
Na^+^	−0.1003	0.0245	−0.4386	0.1445	0.2822	*0.8226*
K^+^	0.1121	0.2247	0.4446	−0.0889	0.3396	*0.7029*
% cumulative explained variation	46.2	67.4	91.0	96.1	98.7	100

Factorial load value above 0.60 was adopted arbitrarily, and therefore these values are italics in the table

The concentration of calcium ions in the examined natural lake part (Table 2) was similar to the results of Grochowska and Tandyrak (2009) and other Polish lakes, in which Ca concentrations oscillates in the range of 23–75 mg/L (Kolada et al. 2005). Water purification processes in the canal via the lake system were clearly visible, e.g. in the section from Augustów to Przewięź. Three lakes with large surfaces and volumes in this section (Fig. 1) decreased the concentration of many dissolved mineral compounds, as shown by the lower electrolytic conductivity (Fig. 2), and also organic compounds (Fig. 3). We can safely say that the lakes included in a semi-natural water system acted as cleansing agents because they immobilized (detoxified) pollution, e.g. sulphate(VI) and chloride ions (Fig. 4). Therefore, it is very important to remember to include the greatest possible number of lakes with a large surface area and volume in designing semi-natural water systems in the future. Our results show it is significant in the functioning of such ecosystems and their biogeochemical processes.

## Conclusions

1. Lake waters in the examined semi-natural water system act as cleansing agents, improving the quality of water in the canal.
2. Streamflow quantity and velocity of river waters flowing into the canal did not influence the physico-chemical properties of waters in the semi-natural water system.
3. River waters poor in organic matter (TOC < 10.0 mg/L) which directly supply the semi-natural water system increase the levels of many tested parameters (EC, pH, TC, TN, SO_4_^2−^ and Cl^−^) in the canal waters.
4. Rivers rich in organic matter (TOC > 10.0 mg/L) which directly supply canal waters result in improved water quality in the semi-natural water system by decreasing in concentrations of nutrients as well as sulphate(VI) and chloride ions.
